# Enhancing gastric cancer conventional chemotherapy effects by triple angiokinase inhibitor nintedanib in preclinical models

**DOI:** 10.3389/fonc.2023.1145999

**Published:** 2023-05-10

**Authors:** Niranjan Awasthi, Margaret A. Schwarz, Quinn Kaurich, Changhua Zhang, Frank Hilberg, Roderich E. Schwarz

**Affiliations:** ^1^ Department of Surgery, Indiana University School of Medicine, South Bend, IN, United States; ^2^ Harper Cancer Research Institute. University of Notre Dame, Notre Dame, IN, United States; ^3^ Department of Pediatrics, Indiana University School of Medicine, South Bend, IN, United States; ^4^ Digestive Diseases Center, The Seventh Affiliated Hospital, Sun Yat-sen University, Shenzhen, Guangdong, China; ^5^ Department of Pharmacology, Boehringer Ingelheim Regional Center Vienna, Vienna, Austria; ^6^ Roswell Park Comprehensive Cancer Center, Buffalo, NY, United States

**Keywords:** gastric cancer, chemotherapy, nintedanib, angiogenesis, combination therapy

## Abstract

**Background:**

Gastric adenocarcinoma (GAC) is the fourth leading cause of cancer death worldwide. Systemic chemotherapy is a preferred treatment option for advanced and recurrent GAC, but response rates and survival prolongation remain limited. Tumor angiogenesis plays a critical role in GAC growth, invasion and metastasis. We investigated the antitumor efficacy of nintedanib, a potent triple angiokinase inhibitor for VEGFR-1/2/3, PDGFR-α/β and FGFR-1/2/3, alone or in combination with chemotherapy, in preclinical models of GAC.

**Methods:**

Animal survival studies were performed in peritoneal dissemination xenografts in NOD/SCID mice using human GAC cell lines MKN-45 and KATO-III. Tumor growth inhibition studies were performed in subcutaneous xenografts in NOD/SCID mice using human GAC cell lines MKN-45 and SNU-5. The mechanistic evaluation involved Immunohistochemistry analyses in tumor tissues obtained from subcutaneous xenografts. *In vitro* cell viability assays were performed using a colorimetric WST-1 reagent.

**Results:**

In MKN-45 GAC cell-derived peritoneal dissemination xenografts, animal survival was improved by nintedanib (33%), docetaxel (100%) and irinotecan (181%), while oxaliplatin, 5-FU and epirubicin had no effect. The addition of nintedanib to docetaxel (157%) or irinotecan (214%) led to a further extension in animal survival. In KATO-III GAC cell-derived xenografts carrying *FGFR2* gene amplification, nintedanib extended survival by 209%. Again, the addition of nintedanib further enhanced the animal survival benefits of docetaxel (273%) and irinotecan (332%). In MKN-45 subcutaneous xenografts, nintedanib, epirubicin, docetaxel and irinotecan reduced tumor growth (range: 68-87%), while 5-FU and oxaliplatin had a smaller effect (40%). Nintedanib addition to all chemotherapeutics demonstrated a further reduction in tumor growth. Subcutaneous tumor analysis revealed that nintedanib attenuated tumor cell proliferation, reduced tumor vasculature and increased tumor cell death.

**Conclusion:**

Nintedanib showed notable antitumor efficacy and significantly improved taxane or irinotecan chemotherapy responses. These findings indicate that nintedanib, alone and in combination with a taxane or irinotecan, has the potential for improving clinical GAC therapy.

## Introduction

Gastric adenocarcinoma (GAC) is the fifth most common cancer and the fourth leading cause of cancer death worldwide (1). For primary metastatic or recurrent GAC, combination chemotherapy regimens lead to a small but clinically significant survival benefit, but median survival remains less than a year (2–6). The triple combination chemotherapy regimen FLOT (5-FU/leucovorin, oxaliplatin and docetaxel) demonstrated better overall survival (OS, 50 months) compared with the ECF/ECX (epirubicin, cisplatin, fluorouracil or capecitabine) group (35 months) as a perioperative therapy for GAC patients with locally advanced, resectable tumor (7). Thus, the FLOT regimen is now the standard regimen for a perioperative strategy of resectable GAC patients and is widely utilized for metastatic GAC, too. Meta-analyses indicate that GAC patients’ survival can be improved by 2^nd^-line therapy after failing 1^st^-line chemotherapy (8, 9). The 2^nd^-line therapy for GAC patients usually resorts to cytotoxic chemotherapy agents taxanes and irinotecan or the two molecular targeted agents trastuzumab and ramucirumab (10). The median OS of GAC patients receiving 2^nd^-line therapy ranges from 3.6 to 10.9 months (11–13). Due to the low response rates of current standard therapies and the development of chemoresistance and toxicity (14), there is a compelling requirement for novel therapeutic options that can improve the outcomes of GAC.

Tumor angiogenesis is a crucial step in the pathogenesis of GAC, facilitating tumor growth, invasion and metastasis. Thus, targeting tumor angiogenesis has been a well-explored therapeutic approach for GAC (15). Angiogenesis is a complex process involving several growth factors and cytokines such as vascular endothelial growth factor (VEGF), fibroblast growth factor (FGF), platelet-derived growth factor (PDGF), tumor necrosis factor-alpha (TNF-α) and angiopoietins (Ang) (16). VEGF and its receptor 2 (VEGFR2)-mediated angiogenic signaling is the most extensively studied pathway in GAC because high levels of circulating and intratumoral VEGF are correlated with tumor aggressiveness and poor survival (17–19). Bevacizumab, the first agent targeting the VEGF axis, in combination with first-line chemotherapy showed some promising activity in several GAC phase II studies (20) but failed to demonstrate clinical efficacy in phase III (AVAGAST) study (21). Ramucirumab is a VEGFR2 monoclonal antibody, which blocks ligand binding and receptor-mediated pathway activation. Ramucirumab is an approved 2^nd^-line treatment as monotherapy or in combination with paclitaxel for advanced GAC patients who have progressed after 5-FU and/or platinum-based chemotherapy (22, 23). Apart from monoclonal antibodies, several small molecule tyrosine kinase inhibitors (TKIs) targeting VEGF/VEGFR2 signaling were also evaluated in GAC (24). Among TKIs, only apatinib, a selective VEGFR2 inhibitor, demonstrated improved PFS and OS in chemotherapy-refractory advanced GAC patients (25).

Despite several studies evaluating anti-VEGF antiangiogenic therapies, improving OS remains a challenge for advanced GAC patients. The survival benefit of antiangiogenic therapies is short because tumors seem to develop several escape mechanisms including the upregulation of compensatory pathways by other angiogenic growth factors. In GAC, apart from VEGF, aberrant signaling of several other growth factors including FGF, PDGF, and IGF have been reported and correlated with poor prognosis (26). Further, the aberrant activation of these growth factor signaling pathways has been implicated in resistance and escape from anti-VEGF therapy (27), suggesting a possible benefit of multi-target antiangiogenic therapies in GAC. Nintedanib (Nin, **Supplementary Figure S1**) is a multi-TKI that targets the receptor kinase(s) of VEGF (IC_50_ 13–34 nM), FGF (IC_50_ 37–108 nM) and PDGF (IC_50_ 59–65 nM). It also targets other kinases such as RET, FLT-3 and Src in the low nanomolar range. In preclinical studies, nintedanib showed antitumor activity in several tumor types (28). In clinical studies, nintedanib combination with chemotherapy showed promising antitumor activity where other antiangiogenic agents failed to show a response suggesting that nintedanib might be superior in such settings (29, 30). Nintedanib is an approved treatment for non-small cell lung cancer (NSCLC) as well as idiopathic pulmonary fibrosis (30, 31).

Since several cytotoxic chemotherapeutic drugs generate only a moderate clinical response in GAC, there appears to be room for improvement in their efficacy by targeted agents. Multitarget antiangiogenic agents such as nintedanib may be more efficacious than single-target agents such as ramucirumab and apatinib but have not been widely explored in GAC, especially in combination with traditionally used chemotherapy drugs. Therefore, this preclinical study determined the most efficacious chemotherapeutics together with the antitumor activity of triple angiokinase inhibitor nintedanib in search for therapeutic combinations with enhanced antitumor efficacy in GAC.

## Materials and methods

### Cell culture and reagents

The human GAC cell lines KATO-III and SNU-5 were purchased from the American Type Culture Collection (ATCC, Rockville, MD). Human GAC cell line MKN-45 was purchased from Creative Bioarray (Shirley, NY). Cell lines were authenticated by ATCC (KATO-III, SNU-5) or Creative Bioarray (MKN-45) and were routinely screened to ensure the absence of mycoplasma contamination (InvivoGen, San Diego, CA). The characteristics of these GAC cell lines are presented in **Supplementary Table 1**. Cells were cultured in RPMI 1640 medium (Sigma Chemical Co. St. Louis, MO) containing 10% or 20% FBS and maintained at 37°C in a humidified incubator with 5% CO_2_ and 95% air. Human gastric fibroblasts were purchased from ScienCell Research Laboratories (Carlsbad, CA) and cultured in a fibroblast specialty medium. Cytotoxic agents 5-FU, epirubicin (Epi), oxaliplatin (Oxa), docetaxel (Doc) and irinotecan (Iri) were purchased from the pharmacy at the Goshen Center for Cancer Care (Goshen, IN). Nintedanib was obtained from Boehringer Ingelheim Pharmaceuticals. The cell proliferation reagent WST-1 was purchased from Sigma-Aldrich.

### Cell proliferation assay

*In vitro* cell proliferation assays were executed by adding the colorimetric WST-1 reagent. Four to five thousand cells were plated in each well of a 96-well plate in the regular growth medium, which was replaced after 16 hours with a 2% FBS-containing medium. The cells were treated with nintedanib, 5-FU, epirubicin, oxaliplatin, docetaxel and irinotecan, and incubated for 72 hours. Following the incubation, WST-1 reagent (10 μl) was added to each well. The cells were incubated for an additional 2 hours at 37°C. After incubation, the absorbance at 450 nm was measured using a microplate reader.

### Tumor implant and *in vivo* tumor growth experiment

Animal experiments were performed following the Institutional Animal Care and Use Committee (IACUC) protocol approved by the Indiana University School of Medicine (South Bend, IN). Six-week-old female nonobese diabetic/severe combined immunodeficient (NOD/SCID) mice were subcutaneously injected with human GAC MKN-45 cells (7.5 x 10^6^) or SNU-5 cells (10x10^6^). Ten days after tumor cell injection, all mice had a measurable tumor. Mice were then randomized (n=5 per group) to receive PBS (control), nintedanib (25 mg/kg, 5x wk), 5-FU (50 mg/kg, 2x wk), epirubicin (1 mg/kg, 2x wk), oxaliplatin (5 mg/kg, 2x wk), docetaxel (2 mg/kg, 2x wk) or irinotecan (10 mg/kg, 1x wk), for two weeks as previously described (32). The doses of nintedanib and chemotherapy agents were selected based on their clinically equivalent, safe and effective dose range described in the literature. Tumor size was measured twice per week and tumor volume was calculated using the formula V=1/2 (L x W^2^), L=length and W=width.

### Animal survival analysis

Animal survival studies were performed using 6-week-old female NOD/SCID mice as previously described (33). Briefly, the mice were intraperitoneally injected with MKN-45 cells (10x10^6^) or KATO-III cells (10x10^6^) and ten days after tumor cell injection, mice were randomized (n=6 to 8 per group) to receive PBS (control), nintedanib (25 mg/kg, 5x wk), 5-FU (50 mg/kg, 2x wk), epirubicin (1 mg/kg, 2x wk), oxaliplatin (5 mg/kg, 2x wk), docetaxel (2 mg/kg, 2x wk) or irinotecan (10 mg/kg, 1x wk), for next two weeks. The experimental procedure of animal survival experiments has been presented in **Supplementary Figure S2**. Animals were evaluated daily for any drug-related toxicities. After completion of treatment, mice were monitored daily and euthanized when moribund according to the predefined criteria, including sudden weight loss or gain (>15%), lethargy, inability to remain upright, and lack of strength (34). Animal survival was determined from the first day of treatment until death.

### Immunohistochemistry and immunofluorescence analysis

Subcutaneous tumors were fixed in 4% paraformaldehyde, dehydrated with graded ethanol series (25%, 50%, 70%, 95% and 100%), embedded in paraffin and sectioned. The tumor sections (5 μm) were then deparaffinized with xylene and rehydrated through a graded ethanol series (100%, 95%, 70% and 50%) followed by heat-mediated antigen retrieval in citrate buffer. The sections were then blocked by CAS buffer for 20 minutes. The tumor sections were incubated for 20 minutes in CAS blocking buffer followed by overnight incubation at 4°C with 1:200 dilution of primary antibodies against Ki67 (rabbit polyclonal; Abcam, ab15580) or endomucin (rat monoclonal; Millipore Sigma, MAB2624). The tumor sections were washed with PBS and incubated with 1:200 dilution of secondary antibody conjugated with Cy3 (Jackson ImmunoResearch Laboratories, West Grove, PA) at room temperature for 40 minutes to visualize the antigen. Intratumoral apoptosis was analyzed by staining tissue sections with “Apoptag Red *In Situ* Apoptosis Detection Kit” according to the manufacturer’s (Millipore, S7165) instructions. Tissues were then mounted with a solution containing 4’,6-diamidino-2-phenylindole (DAPI) (Invitrogen, Carlsbad, CA). Fluorescence microscopy was used to detect fluorescent signals in five representative high-power field (HPF) per sample using an IX81 Olympus microscope and images were captured with a Hamamatsu Orca digital camera (Hamamatsu Corporation, Bridgewater, NJ) with a DSU spinning confocal unit using cellSens Dimension software (Olympus, Center Valley, PA). All the immunofluorescence experiments were normalized for exposure time.

### Statistical analysis

The two-tailed Student’s t-test (GraphPad Prism 7.0 Software, San Diego, CA) was used to analyze the statistical significance for the individual group comparison. For *in vivo* tumor growth studies, statistical analysis was executed by one-way ANOVA for multiple group comparisons and Student’s t-test for the individual group comparisons. Nonparametric testing with log-rank group comparisons (GraphPad Prism 7.0) was applied for survival study statistics. *In vitro* cell proliferation data are expressed as the mean ± standard deviation. The statistical significance was determined based on the p-value between control and therapy groups (not significant, p>0.05; *p<0.05; **p<0.01; ***p<0.001; ****p<0.0001), and between nintedanib and combination therapy groups (not significant, p>0.05; ^#^p<0.05; ^##^p<0.01; ^###^p<0.001; ^####^p<0.0001). Similar to our recently published methodology about the sample size (35), we used G*Power 3.1 software for the power calculation in animal experiments. We used 6 to 8 mice per group in animal survival experiments and 5 mice per group in subcutaneous tumor growth experiments. With a sample size of 5 to 8 mice per group, a preset α value of 0.05, statistically significant differences in animal survival or tumor size of 40%, and a standard deviation of 20% could be detected at a power of greater than 80%. In this preclinical study, an external validation cohort was not planned.

## Results

### Improvement in animal survival by nintedanib and cytotoxic agents

**Figure 1:**
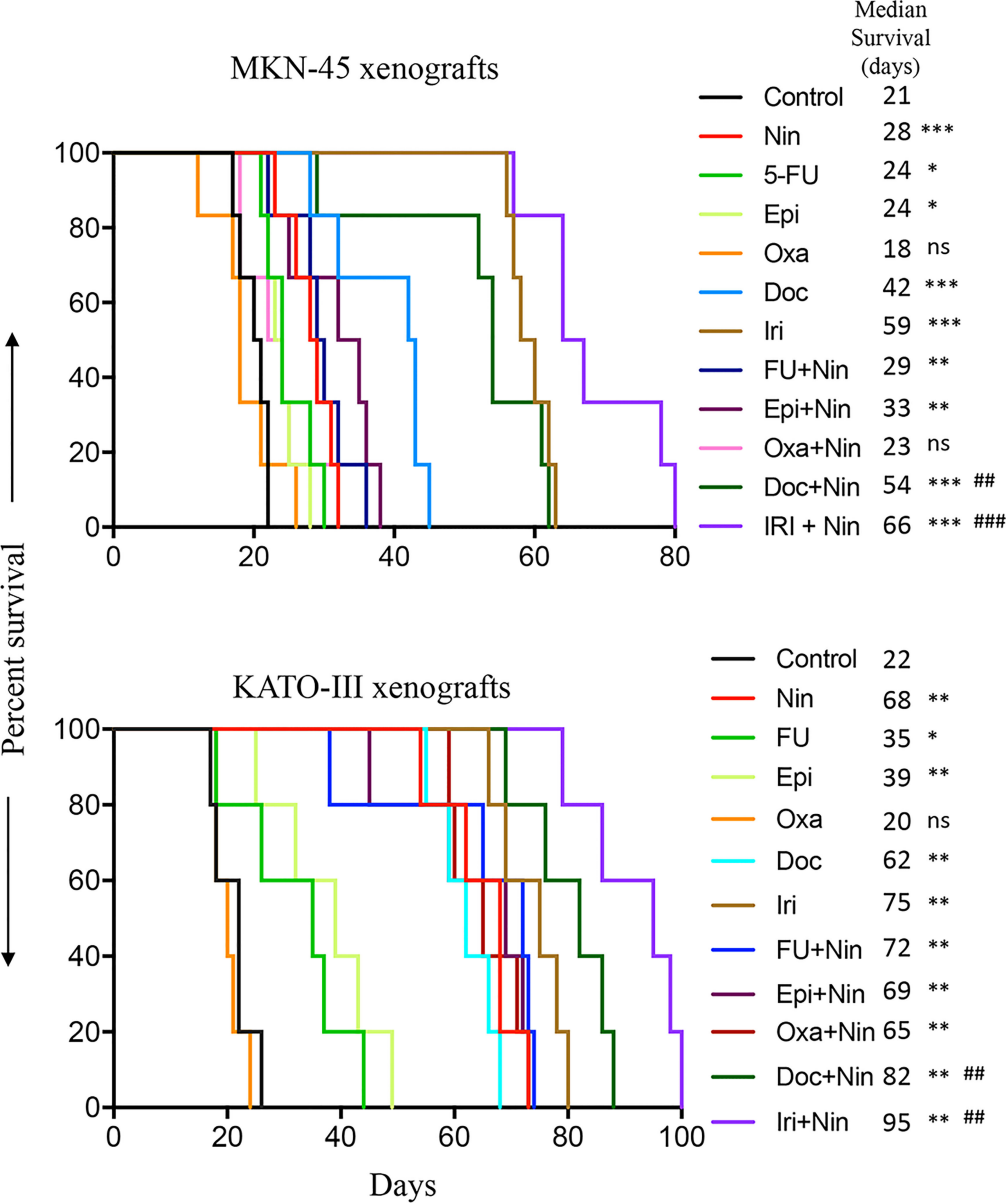
Animal survival benefits of nintedanib and cytotoxic agents. Animal survival analysis in MKN-45 (n=7) and KATO-III (n=5) cell-derived peritoneal dissemination xenografts. Ten days after tumor cell injection, mice were treated with nintedanib, 5-FU, epirubicin, oxaliplatin, docetaxel, irinotecan, or their combinations for two weeks. The curve represents the animal survival time from the start of therapy. Statistical group differences in survival time were calculated using log-rank testing. The statistical significance was determined based on the p-value between control and therapy groups (ns, not significant, p>0.05; *p<0.05; **p<0.01; ***p<0.001), and between nintedanib and combination therapy groups (^##^p<0.01; ^###^p<0.001).

Considering the fact that peritoneal metastasis is a hallmark of advanced GAC, we determined the efficacy of nintedanib and cytotoxic drugs in improving animal survival using the human GAC peritoneal dissemination model with MKN-45 cells (diffuse type, derived from the metastatic site, low *FGFR2* expression). Compared with controls (21 days), animal survival was significantly improved by nintedanib monotherapy (28 days, a 33% increase, p=0.0008). Among cytotoxic agent monotherapy, animal survival was not affected by oxaliplatin (18 days, p=0.75), slightly increased by 5-FU and epirubicin (both 24 days, a 14% increase, p=0.01), and markedly increased by docetaxel (42 days, a 100% increase, p=0.0008) and irinotecan (59 days, a 181% increase, p=0.0008). The addition of nintedanib did not increase survival in oxaliplatin (Oxa+Nin: 23 days, p=0.25), 5-FU (FU+Nin: 29 days, p=0.45) or epirubicin (Epi+Nin: 33 days, p=0.11), compared with nintedanib monotherapy. Importantly, there was a notable increase in animal survival by the addition of nintedanib to docetaxel or irinotecan compared with monotherapies: Doc+Nin (54 days, a 29% increase *vs* Doc, p=0.01; a 93% increase *vs* Nin, p=0.004) and Iri+Nin (66 days, a 12% increase *vs* Iri, p=0.007; a 136% increase *vs* Nin, p=0.0005) (**Figure 1**).

Animal survival was also evaluated in another GAC peritoneal dissemination model using KATO-III cells (diffuse type, derived from the metastatic site, *FGFR2*-amplified). In this experiment, median survival in PBS-treated controls was 22 days. Compared with controls, animal survival was not increased by oxaliplatin (20 days, p=0.49), but strikingly increased by monotherapy with nintedanib (68 days, p=0.002), 5-FU (35 days, p=0.041), epirubicin (39 days, p=0.007), docetaxel (62 days, p=0.002) and irinotecan (75 days, p=0.002). The addition of nintedanib to docetaxel and irinotecan exhibited a further improvement in animal survival: Doc+Nin (82 days, p=0.002 *vs* Doc, p=0.007 *vs* Nin) and Iri+Nin (95 days, p=0.006 *vs* Iri, p=0.002 *vs* Nin). However, nintedanib addition to 5-FU, epirubicin or oxaliplatin did not increase animal survival compared with nintedanib monotherapy (**Figure 1**).

### Tumor growth retardation by nintedanib and cytotoxic agents

**Figure 2:**
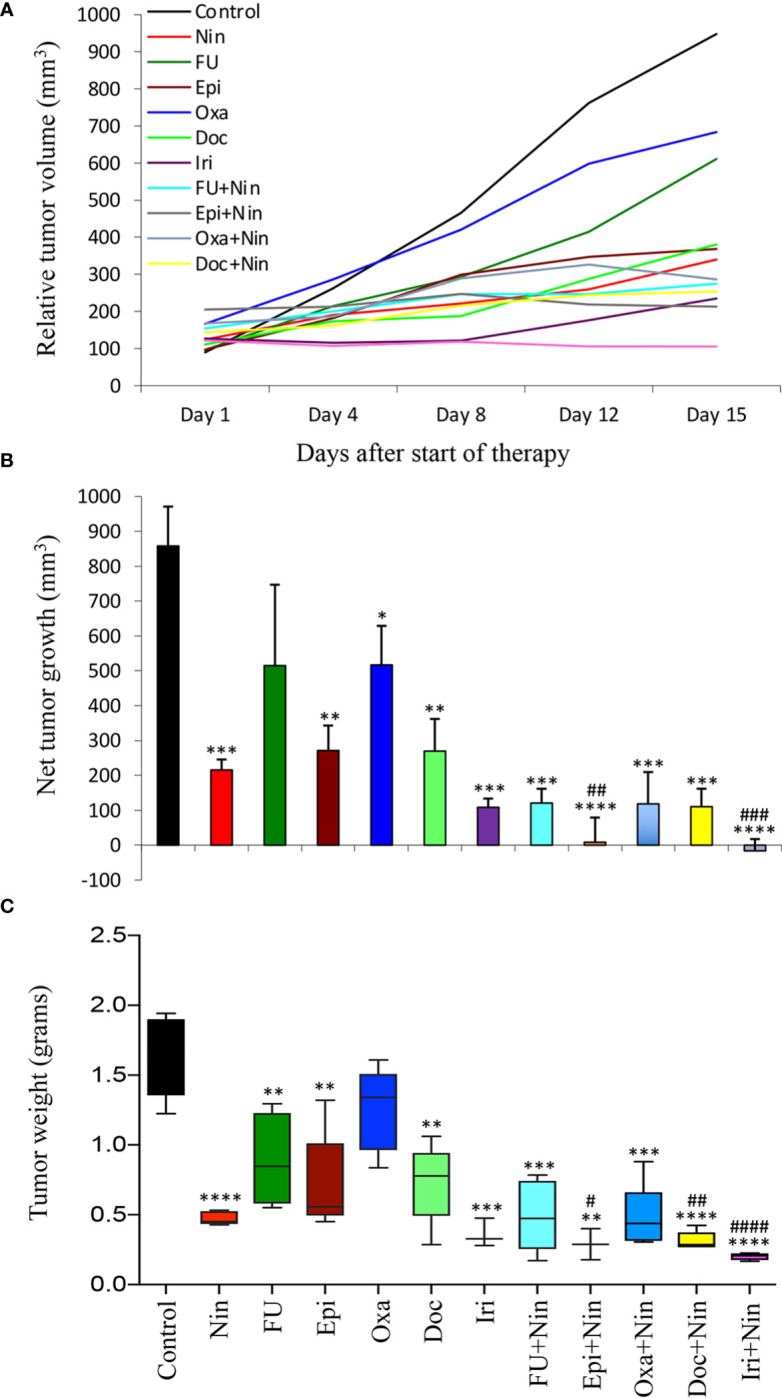
Tumor growth retardation by nintedanib and cytotoxic agents in MKN-45 cell-derived subcutaneous xenografts: Ten days after tumor cell injection, mice were treated with nintedanib, 5-FU, epirubicin, oxaliplatin, docetaxel, irinotecan or their combinations for two weeks. **(A)** Tumor size was measured twice a week during the therapy period using calipers and plotted. **(B)** Net growth in tumor size was calculated by subtracting tumor volume on the first treatment day from that on the final day. **(C)** On the final therapy day, tumors were excised, weighed, and the mean tumor weight was calculated in each group and presented as a Box plot. Student’s t-test was done between control and therapy groups (*p<0.05; **p<0.01; ***p<0.001; ****p<0.0001), and between nintedanib and combination therapy groups (^#^p<0.05; ^##^p<0.01; ^###^p<0.001; ^####^p<0.0001). Data are representative of mean values ± standard deviation from at least 5 mice per group.

Antitumor efficacy of nintedanib and cytotoxic agents was further evaluated in subcutaneous xenografts using MKN-45 cells. Compared with rapid growth in tumor size in the control (PBS-treated) mice, single-agent 5-FU and oxaliplatin resulted in a small reduction in tumor growth, while nintedanib, epirubicin, docetaxel and irinotecan exhibited a marked tumor growth retardation. The combination of nintedanib with cytotoxic agents resulted in a remarkable synergistic tumor growth retardation effect (**Figure 2A**). Net increase in tumor volume, compared to controls (858 mm^3^), was 216 mm^3^ (nintedanib), 515 mm^3^ (5-FU), 271 mm^3^ (epirubicin), 517 mm^3^ (oxaliplatin), 270 mm^3^ (docetaxel), 108 mm^3^ (irinotecan), 121 mm^3^ (5-FU plus nintedanib), 8 mm^3^ (epirubicin plus nintedanib), 118 mm^3^ (oxaliplatin plus nintedanib), 110 mm^3^ (docetaxel plus nintedanib) and -16 mm^3^ (tumor regression, irinotecan plus nintedanib), respectively (**Figure 2B**). At the end of two weeks of therapy, the mean tumor weight in the control group was 1.61 g, which was reduced by the nintedanib treatment to 0.47g. The mean tumor weight in the single-agent chemotherapy groups was in the range of 0.35-1.23 g. In the nintedanib plus chemotherapy combination treatment groups, the mean tumor weight was in the range of 0.20-0.44 g (**Figure 2C**). There was no significant difference in the body weight in the control or therapy group animals indicating that there was no apparent treatment-related toxicity (**Supplementary Figure S3**).

**Figure 3:**
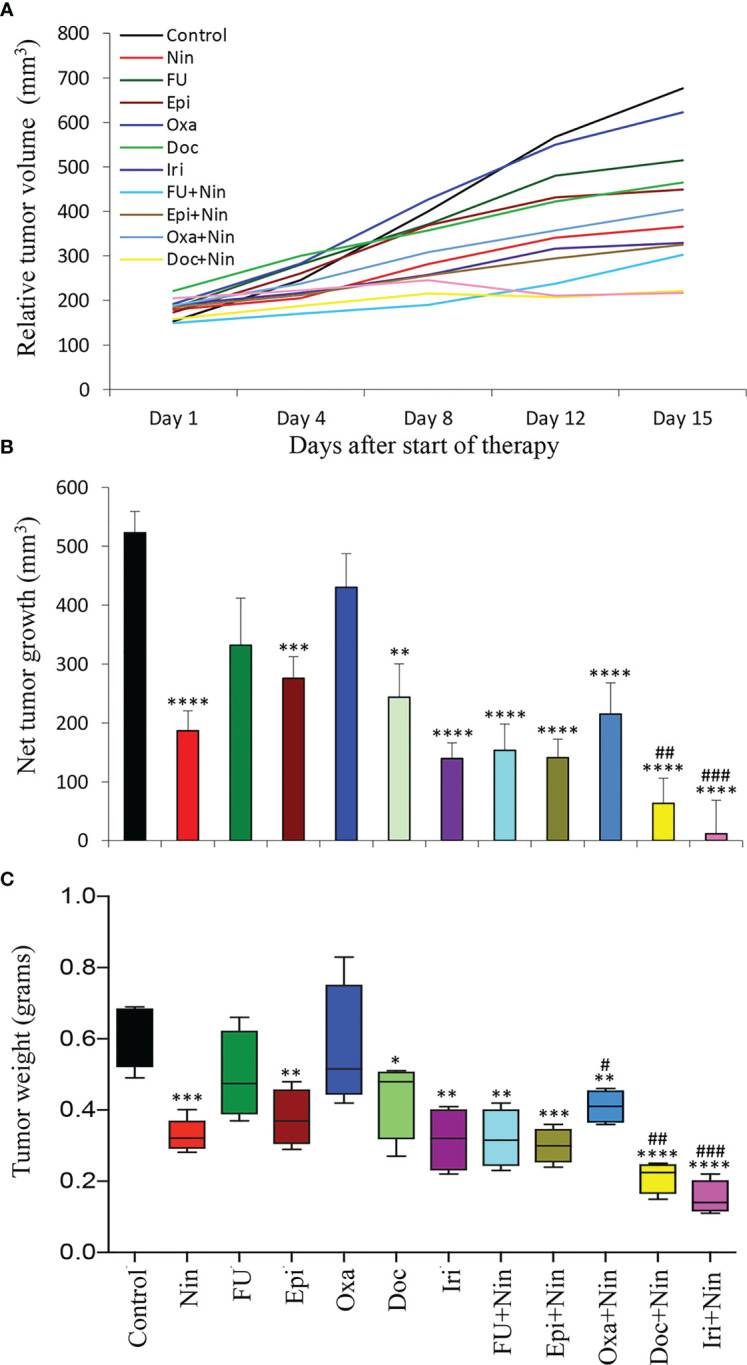
Tumor growth retardation by nintedanib and cytotoxic agents in SNU-5 cell-derived subcutaneous xenografts: Ten days after tumor cell injection, mice were treated with nintedanib, 5-FU, epirubicin, oxaliplatin, docetaxel, irinotecan or their combinations for two weeks. **(A)** Tumor size was measured twice a week during the therapy period using calipers and plotted. **(B)** Net growth in tumor size was calculated by subtracting tumor volume on the first treatment day from that on the final day. **(C)** On the final therapy day, tumors were excised, weighed, and the mean tumor weight was calculated in each group and presented as a Box plot. Student’s t-test was done between control and therapy groups (*p<0.05; **p<0.01; ***p<0.001; ****p<0.0001), and between nintedanib and combination therapy groups (^#^p<0.05; ^##^p<0.01; ^###^p<0.001). Data are representative of mean values ± standard deviation from at least 5 mice per group.

In human GAC subcutaneous xenografts using SNU-5 cells (derived from malignant ascites), single-agent 5-FU and oxaliplatin had a small effect, while a significant reduction in tumor growth was observed with nintedanib, epirubicin, docetaxel and irinotecan monotherapy and nintedanib combination with all cytotoxic agents had a synergistic response (**Figure 3A**). This study demonstrated that compared to controls (523 mm^3^), the net tumor growth was 187 mm^3^ (nintedanib), 332 mm^3^ (5-FU), 276 mm^3^ (epirubicin), 430 mm^3^ (oxaliplatin), 243 mm^3^ (docetaxel), 139 mm^3^ (irinotecan), 153 mm^3^ (5-FU plus nintedanib), 141 mm^3^ (epirubicin plus nintedanib), 215 mm^3^ (oxaliplatin plus nintedanib), 63 mm^3^ (docetaxel plus nintedanib) and 12 mm^3^ (irinotecan plus nintedanib), respectively (**Figure 3B**). After two weeks of therapy, the mean tumor weight correlated with tumor growth inhibition data; it was 0.61 g in the control and 0.33 g in nintedanib. The mean tumor weight by single-agent chemotherapy was in the range of 0.31-0.55 g, while it further decreased by the nintedanib plus chemotherapy combination therapy exhibiting tumor weights in the range of 0.15-0.41 g (**Figure 3C**). Like the MKN-45 xenograft study, there was no treatment-related toxicity in different therapy groups (**Supplementary Figure S3**).

### Effects of nintedanib and cytotoxic agents on tumor cell proliferation and tumor vasculature

**Figure 4:**
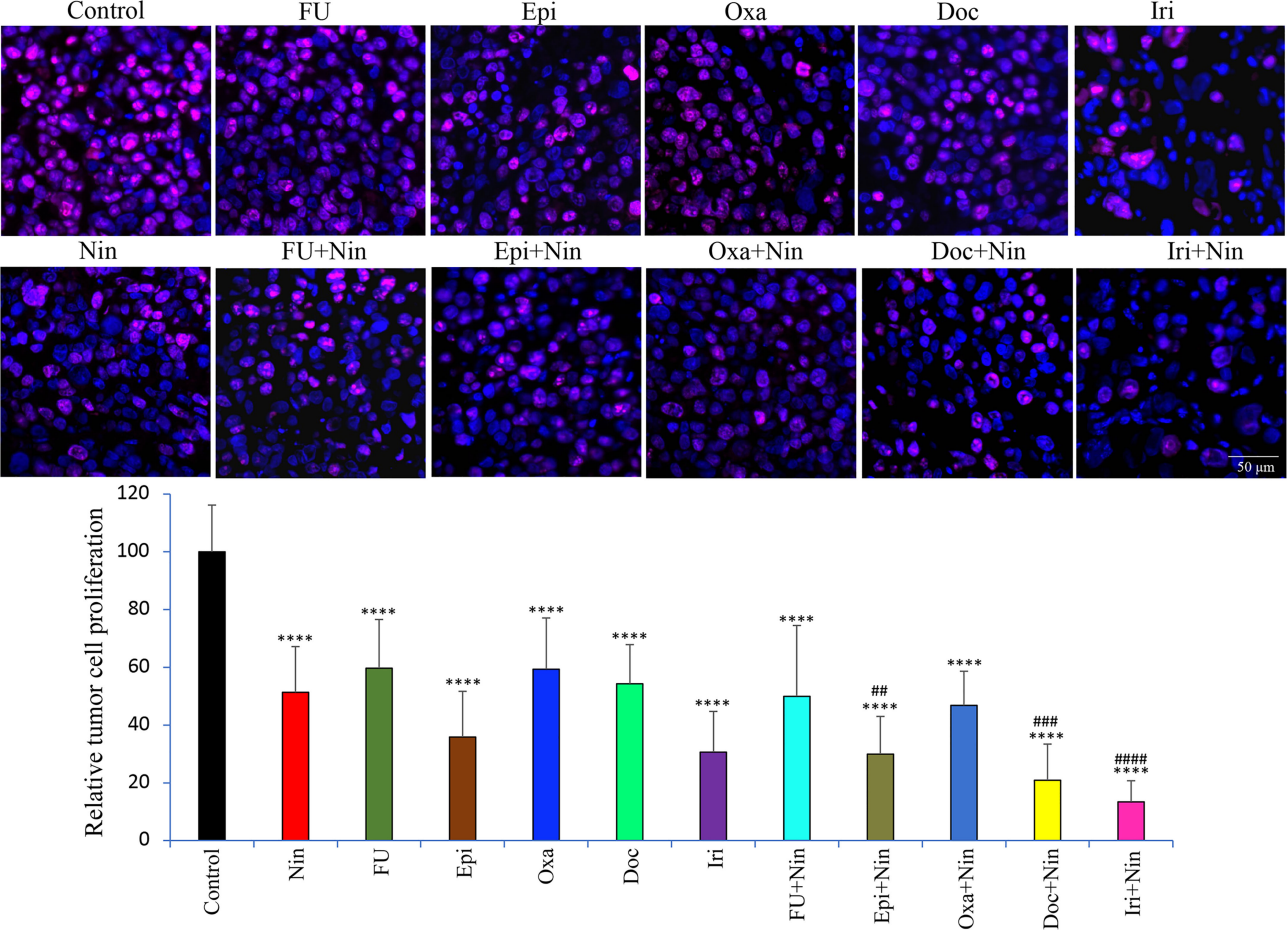
Effect of nintedanib and cytotoxic agents on tumor cell proliferation. Tumor sections obtained from the MKN-45 subcutaneous xenograft study after two weeks of treatment were used for the IHC analysis. Tissue sections were immunostained with Ki67 antibody and photographed under a fluorescent microscope. Ki67-positive cells were counted in five different high-power fields. The upper panel depicts merged images of cell nuclei stained with Ki67 (red) and DAPI (blue) illustrated at 20X magnification. Student’s t-test was done between control and therapy groups (****p<0.0001), and between nintedanib and combination therapy groups (^##^p<0.01; ^###^p<0.001; ^####^p<0.0001). The data are expressed as the mean ± standard deviation.

We next investigated the biological impact of nintedanib and cytotoxic agents on GAC tissues. Ki67 staining to examine tumor cell proliferation in MKN-45 subcutaneous xenografts demonstrated that monotherapy with nintedanib and all cytotoxic agents reduced tumor cell proliferation, while combinations of nintedanib with cytotoxic agents were more effective than single agents. Again, the combination of nintedanib with docetaxel or irinotecan exhibited maximum inhibition in tumor cell proliferation. Compared to controls (100%), the intratumoral proliferative index, measured by calculating Ki67 positive cells over the total number of cells per HPF, was as follows: Nin (51.3±15.8%), 5-FU (59.7±16.9%), Epi (35.8±15.8%), Oxa (59.3±17.7%), Doc (54.3±13.6%), Iri (30.6±14.1%), 5-FU+Nin (49.9±24.5%), Epi+Nin (29.9±13%), Oxa+Nin (46.8±11.8%), Doc+Nin (20.9±12.5%) and Iri+Nin (13.4±7.3%) (**Figure 4**).

**Figure 5:**
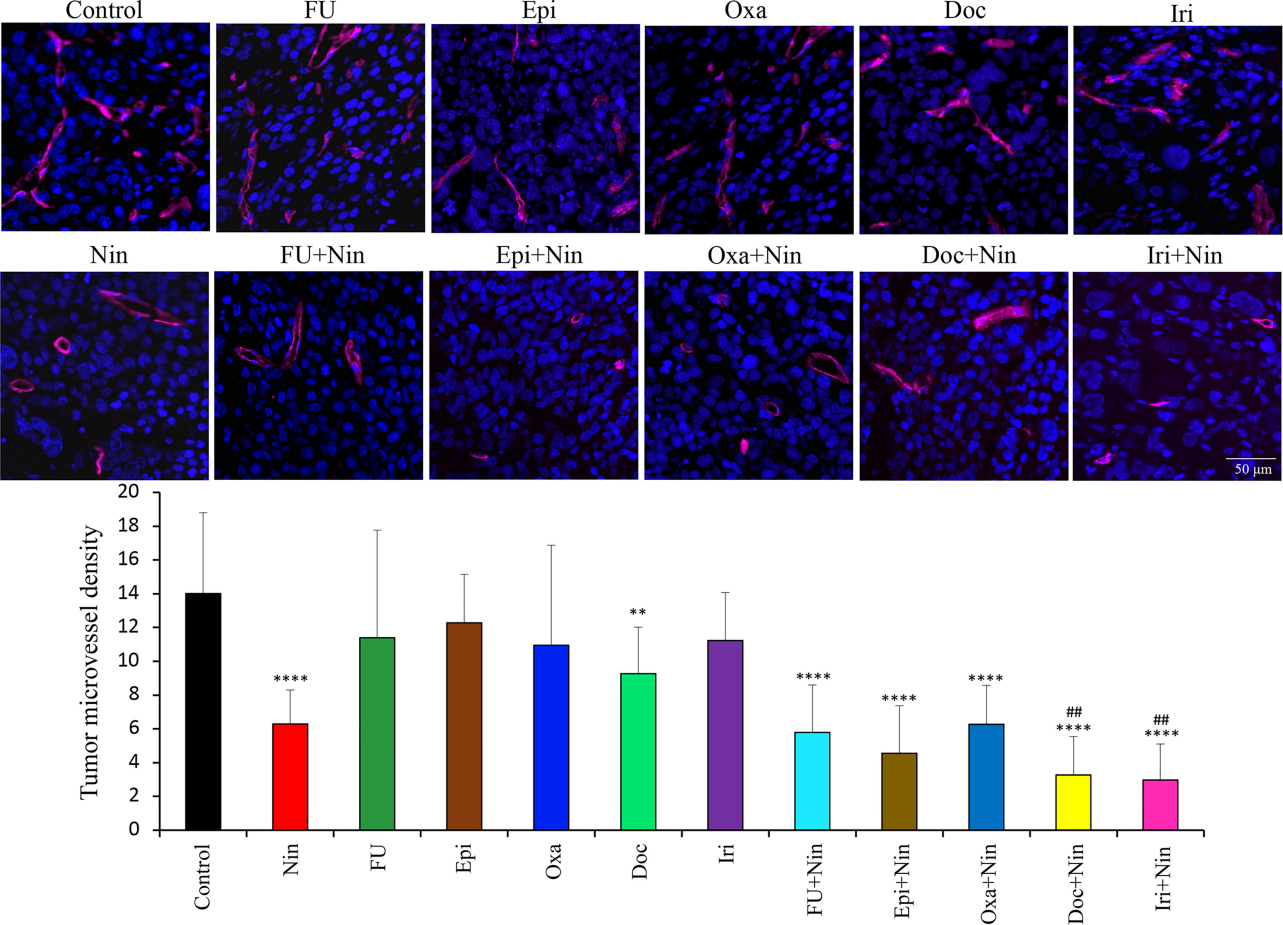
Effect of nintedanib and cytotoxic agents on microvessel density. Tumor sections obtained from the MKN-45 subcutaneous xenograft study after two weeks of treatment were used for evaluating intratumoral microvessel density. Tumor sections were stained with an anti-endomucin antibody and slides were photographed under a fluorescent microscope. Endomucin-positive vessels were counted within five different HPF in a blinded manner. The upper panel depicts merged images of endomucin-positive microvessel (red) and cell nuclei (DAPI, blue) illustrated at 20X magnification. Student’s t-test was done between control and therapy groups (**p<0.01; ****p<0.0001), and between nintedanib and combination therapy groups (^##^p<0.01). The data are expressed as the mean ± standard deviation.

Assessment of the effects of therapies on tumor vasculature by endomucin staining revealed that nintedanib caused a remarkable decrease in microvessel density, while cytotoxic agents exhibited no significant change. Nintedanib combination with cytotoxic agents also led to a reduction in microvessel density compared with controls but this was not significantly greater than nintedanib monotherapy. Mean microvessel counts were as follows: Control (14±4.8), Nin (6.3±2), 5-FU (11.4±6.4), Epi (12.3±2.9), Oxa (10.9±5.9), Doc (9.3±2.8), Iri (11.2±2.8), 5-FU+Nin (5.8±2.8), Epi+Nin (4.6±2.8), Oxa+Nin (6.3±2.3), Doc+Nin (3.2±2.3) and Iri+Nin (2.9±2.1) (**Figure 5**).

### Effects of nintedanib and cytotoxic agents on tumor cell apoptosis

**Figure 6:**
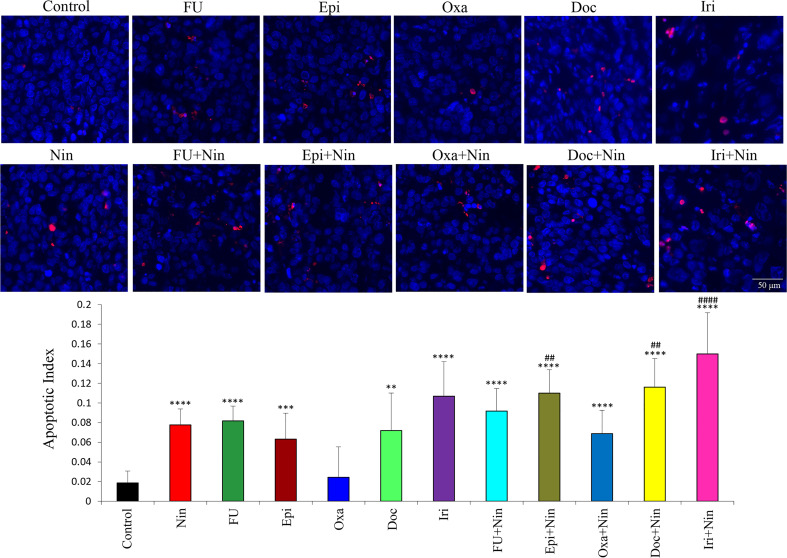
Effect of nintedanib and cytotoxic agents on tumor cell apoptosis. Tumor tissue sections obtained from the MKN-45 subcutaneous xenograft study after two weeks of treatment were stained with the TUNEL procedure. TUNEL-positive apoptotic cells were counted in five different high-power fields and slides were photographed under a fluorescent microscope. Student’s t-test was done between control and therapy groups (**p<0.01; ***p<0.001; ****p<0.0001), and between nintedanib and combination therapy groups (^##^p<0.01; ^####^p<0.0001). The data are expressed as the mean ± standard deviation.

An investigation of the impact of nintedanib and cytotoxic therapies on tumor cell apoptosis in MKN-45 subcutaneous tumor tissues demonstrated that compared with controls (apoptosis index: 0.018), nintedanib was effective in inducing apoptosis (0.077). Except for oxaliplatin (0.024) other chemotherapy drugs also induced apoptosis in the following order: epirubicin (0.063), docetaxel (0.072), 5-FU (0.082) and irinotecan (0.107). Increase in intratumoral apoptosis in combinations of nintedanib with epirubicin (0.109), docetaxel (0.116) and irinotecan (0.15) were significantly higher than in single-agent treatment groups (**Figure 6**).

### Effects of nintedanib and chemotherapy agents on *in vitro* GAC cell proliferation

**Figure 7:**
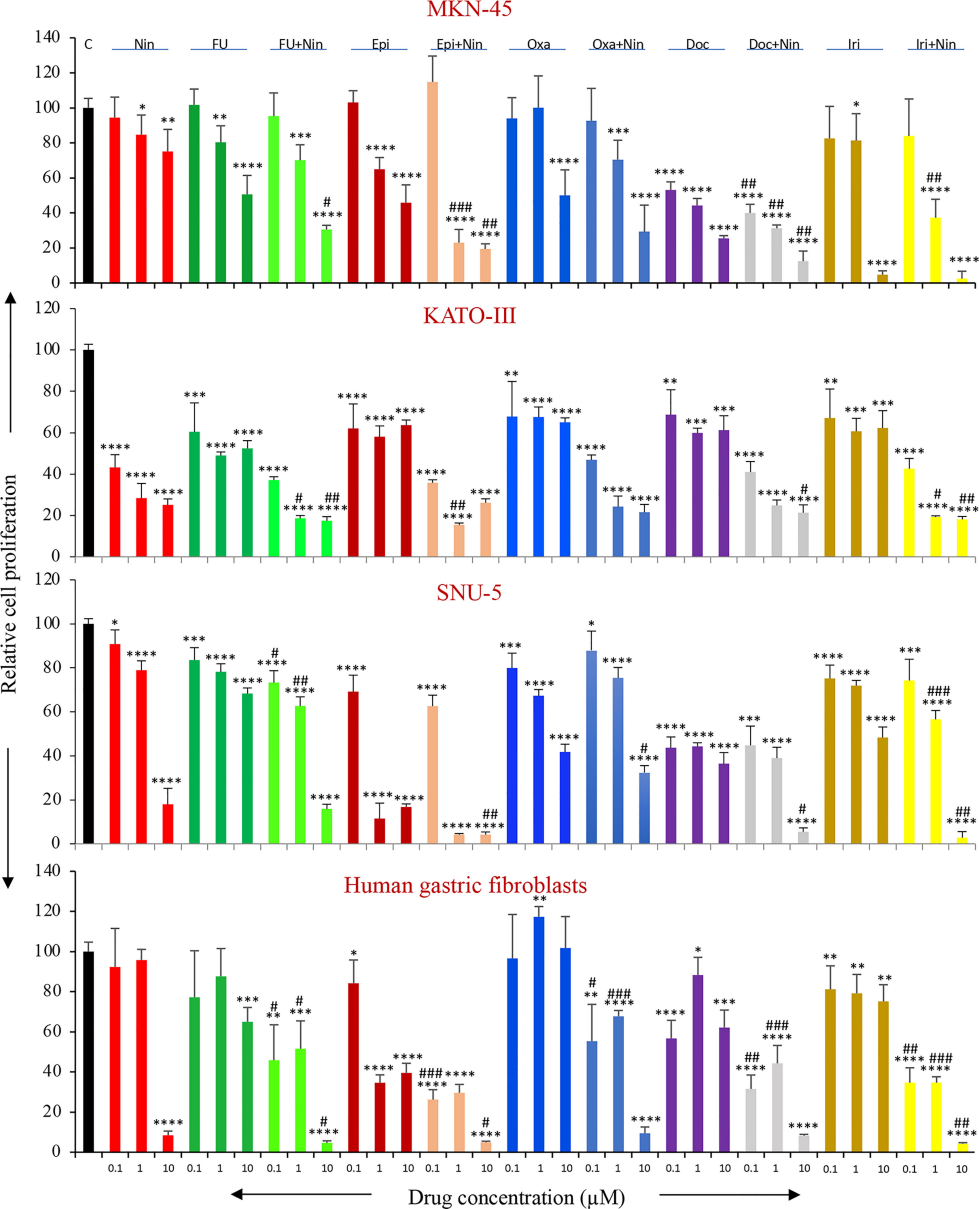
*In vitro* cell proliferation inhibition by nintedanib and cytotoxic agents. GAC cells (MKN-45, KATO-III and SNU-5) and human gastric fibroblasts were plated on 96-well plates and treated with nintedanib, 5-FU, epirubicin, oxaliplatin, docetaxel and irinotecan. After 72-hour incubation, WST-1 reagent (10 μl) was added to each well followed by additional incubation for 2 hours. The absorbance at 450 nm was measured using a microplate reader. The resulting number of viable cells was calculated by measuring the absorbance of color produced in each well. Student’s t-test was done between control and therapy groups (*p<0.05; **p<0.01; ***p<0.001; ****p<0.0001), and between nintedanib or respective cytotoxic therapy and combination therapy groups (^#^p<0.05; ^##^p<0.01; ^###^p<0.001). The data are the mean ± SD of quadruplicate determinations.

Human GAC epithelial cells with different oncogenic mutations (MKN-45, KATO-III, SNU-5) and gastric fibroblasts were tested for growth-inhibitory effects by nintedanib and cytotoxic agents. Nintedanib had a significant growth inhibitory effect on these cell lines. Reduction in cell proliferation by nintedanib at 100 nM, 1 μM and 10 μM concentrations were 6%, 21%, 25% (MKN-45); 57%, 72%, 75% (KATO-III); 9%, 21%, 82% (SNU-5) and 8%, 4%, 92% (gastric fibroblasts) (**Figure 7**).

All cytotoxic drugs exhibited dose-dependent inhibition in the proliferation of GAC cells proliferation. Inhibition in cell proliferation at a medium dose (1 μM) in MKN-45, KATO-III, SNU-5 and gastric fibroblast cells was 20%, 51%, 22%, 12% (5-FU); 35%, 42%, 89%, 65% (Epi); 0%, 32%, 33%, 0% (Oxa); 56%, 40%, 56%, 12% (Doc); 19%, 39%, 28%, 21% (Iri) (**Figure 7**). Notably, the combination of nintedanib with cytotoxic agents demonstrated additive effects, except for Oxa+Nin in SNU-5 cells, and inhibition in GAC cell proliferation at a medium dose (1 μM) in MKN-45, KATO-III, SNU-5 and gastric fibroblasts was 30%, 81%, 37%, 42% (5-FU+Nin); 77%, 85%, 96%, 70% (Epi+Nin); 30%, 76%, 25%, 32% (Oxa+Nin); 69%, 75%, 61%, 56% (Doc+Nin); 63%, 81%, 43%, 65% (Iri+Nin) (**Figure 7**).

## Discussion

Chemotherapy remains the mainstay treatment for primary metastatic or recurrent GAC. However, cytotoxic regimens have to date all demonstrated limitations in clinical response and survival. Advancement in molecular profiling has paved the way for several novel therapeutic options for GAC patients with targetable oncogenic pathways including *HER2* amplification, *PIK3CA* mutation, JAK pathway, epithelial-mesenchymal transition, RTK amplification and angiogenesis (36). GAC development, relapse and metastatic dissemination critically depend on angiogenesis, which provides required nutrients, growth factors and oxygen. Tumor angiogenesis in GAC is regulated by several growth factors, growth factor receptors and cytokines (15). The FDA approval of molecular targeted agents, trastuzumab and ramucirumab, for advanced GAC, indicates the potential for growth-inhibitory and antiangiogenic treatments for improving GAC clinical therapy. Several studies indicated challenges in improving OS by anti-VEGF therapies in advanced GAC patients due to the implication of compensatory pathways by other angiogenic growth factors including FGF, PDGF, and IGF (26). These growth factor signaling pathways play a critical role in limiting the therapeutic potential of single VEGF-targeted therapy (27), indicating a potential for the multi-target antiangiogenic approach in GAC. Based on the triple angiokinase activity of nintedanib at low doses, we explored its antitumor efficacy as monotherapy and its potential to improve the response of conventional chemotherapy agents in diverse preclinical models of GAC.

Peritoneal metastasis is the most frequent form of metastasis in advanced GAC, and it is associated with poor prognosis (37, 38). We established two peritoneal dissemination xenograft models using human MKN-45 cells and KATO-III cells that closely resemble the clinical GAC progression pattern (39). Nintedanib exhibited noticeable improvement in animal survival in these two models, which was much higher in KATO-III xenografts compared with MKN-45 xenografts. Of note, this survival extension was observed after a limited treatment duration, without any maintenance therapy. Higher animal survival benefits by nintedanib in KATO-III xenografts can be attributed to the fact that KATO-III cells carry *FGFR2* gene amplification (40) rendering it susceptible to nintedanib’s unique targeting profile that includes the FGF-FGFR signaling axis (28). Additionally, nintedanib also demonstrated a marked reduction in tumor growth in GAC cell-derived subcutaneous xenografts.

Gastric cancer is frequently diagnosed in more advanced stages except in Japan and Korea where some screening programs are being implemented. For late-stage or recurred GAC patients, systemic chemotherapy regimens including 5-FU/capecitabine, platinum compounds (cisplatin, oxaliplatin), taxanes (docetaxel, paclitaxel), epirubicin, and irinotecan have shown some benefit but an internationally accepted single standard chemotherapy regimen is still lacking. Among the five commonly used GAC chemotherapy drugs in this study, antitumor efficacy was low for oxaliplatin, moderate for 5-FU and epirubicin, and high for docetaxel and irinotecan, in the peritoneal dissemination xenograft models. In the subcutaneous xenograft models, 5-FU and oxaliplatin were moderately effective while epirubicin, docetaxel and irinotecan were highly effective. Beyond simple dosage and administration frequency, differential tumor responsiveness of various chemotherapy drugs in this study may be dependent on tumor histology, heterogeneity and growth rate. Another possibility for the mechanism of this differential effect of chemotherapy drugs is that the patient-derived cell lines used in these studies were previously exposed with 5-FU, epirubicin and oxaliplatin but not with docetaxel and irinotecan that have an established clinical track record as 2^nd^-line therapy in GAC (10).

Single-agent antiangiogenic therapies have dismal clinical benefits, supporting a combination therapy approach by combining angiogenesis blockade therapy with other conventional therapies, such as immunotherapy, radiotherapy and chemotherapy (41). In our studies, a combination of nintedanib with mechanistically different chemotherapies exhibited a marked increase in antitumor response compared with single-agent therapies. Among all the tested combinations, the antitumor efficacy was highest in the combination of nintedanib with docetaxel and irinotecan. Consistent with our findings, a meta-analysis of randomized controlled trials showed significantly improved survival and anti-tumor activity with the combination of multitarget antiangiogenic agents and taxane-containing chemotherapy in advanced NSCLC (42). Further, the combination of irinotecan with multitarget antiangiogenic drugs has been shown to have a synergistic antitumor response in gastric cancer and colorectal cancer models (39, 43).

Tumor angiogenesis leads to leaky blood vessels, elevated interstitial fluid pressure (IFP) and low blood perfusion in the tumor microenvironment which provides barriers for drug delivery (44, 45). Although the mechanisms for nintedanib-led enhancement in chemotherapy response are not clear, IHC analyses of tumor tissues suggest a decrease in vessel density and an induction in tumor cell apoptosis as likely factors. Other possible mechanisms of nintedanib augmentation of chemotherapy response include a decrease in vessel wall permeability, normalization of tumor vasculature, decrease in IFP and an increase in perfusion (46). If an agent such as nintedanib can mediate similar benefits in experimental GAC therapy, independent from the cytotoxic agent utilized, it presents a promising tool for future GAC therapeutic combinations. Although genomic-directed stratifications and individualized approaches are reasonable thoughts for well-designed clinical trials in GAC, the agents used in this study, whether cytotoxic or in form of the multikinase inhibitor nintedanib, would not easily lend themselves to a mechanistically oriented genomic approach. In fact, since angiogenic activation in GAC progression reflects predominantly activation of autochthonous physiologic mechanisms, it should lend itself to a broader, less restricted therapeutic approach.

The crosstalk between angiogenesis and immunosuppression mechanisms within the tumor microenvironment and the therapeutic potential for the combination of antiangiogenic therapy and immunotherapy are now well-recognized (47–49). Based on the finding of this study, multitarget antiangiogenic combination therapy opens the avenue for the addition of an immunotherapy agent to achieve the best antitumor response in GAC. Although immunotherapy strategies are complicated in murine xenograft settings using human tumor cells, the addition of immunotherapy would be an interesting combination to explore in future studies.

In the background of multiple current clinical attempts for antiangiogenic combinations therapies in GAC, the present preclinical study demonstrates the higher antitumor activity of taxanes and irinotecan among several mechanistically different chemotherapy agents. This study also highlights the remarkable antitumor efficacy of the multitarget antiangiogenic drug nintedanib and a significant additive response of its combination with taxane and irinotecan. Thus, future clinical studies applying taxanes and/or irinotecan as cytotoxic drugs in combination with nintedanib to improve the clinical outcome of advanced GAC patients would appear sensible.

## Data availability statement

The original contributions presented in the study are included in the article/**Supplementary Material**. Further inquiries can be directed to the corresponding author.

## Ethics statement

The animal study was reviewed and approved by The Institutional Animal Care and Use Committee (IACUC) of the Indiana University School of Medicine (South Bend, IN).

## Author contributions

NA: Conceptualization, data curation, formal analysis, supervision, funding acquisition, validation, investigation, visualization, methodology, writing original draft, editing. MS, QK and CZ: Formal analysis, data curation, methodology. FH: Conceptualization, resources, editing. RS: Conceptualization, supervision, editing. All authors contributed to the article and approved the submitted version.
