# Supplementary figures and images for: Enhancing gastric cancer conventional chemotherapy effects by triple angiokinase inhibitor nintedanib in preclinical models
Source: Front Oncol. 2023 May 10;13:1145999. doi: 10.3389/fonc.2023.1145999 (PMC10206228; doi:10.3389/fonc.2023.1145999)

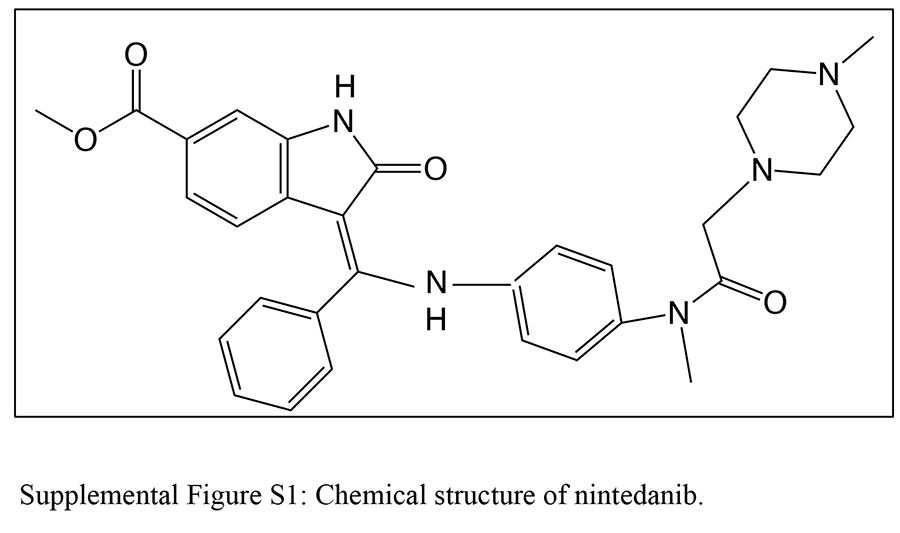

Supplement: Supplementary file 1 [file Image_1.tif]

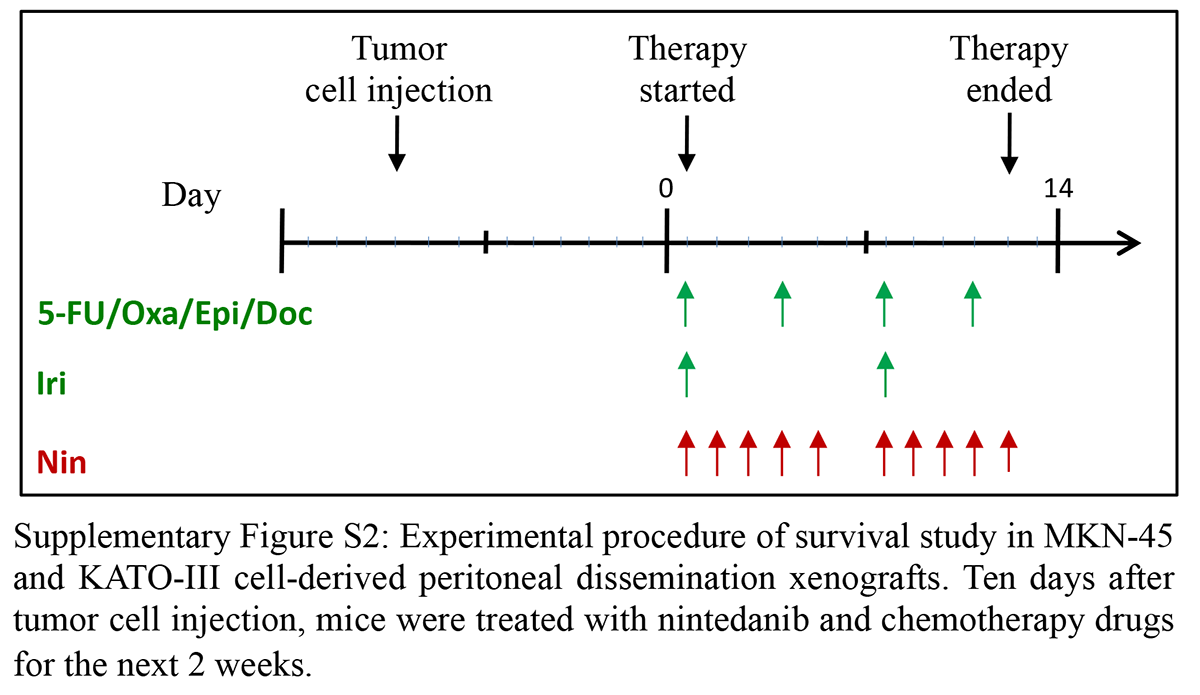

Supplement: Supplementary file 2 [file Image_2.tif]

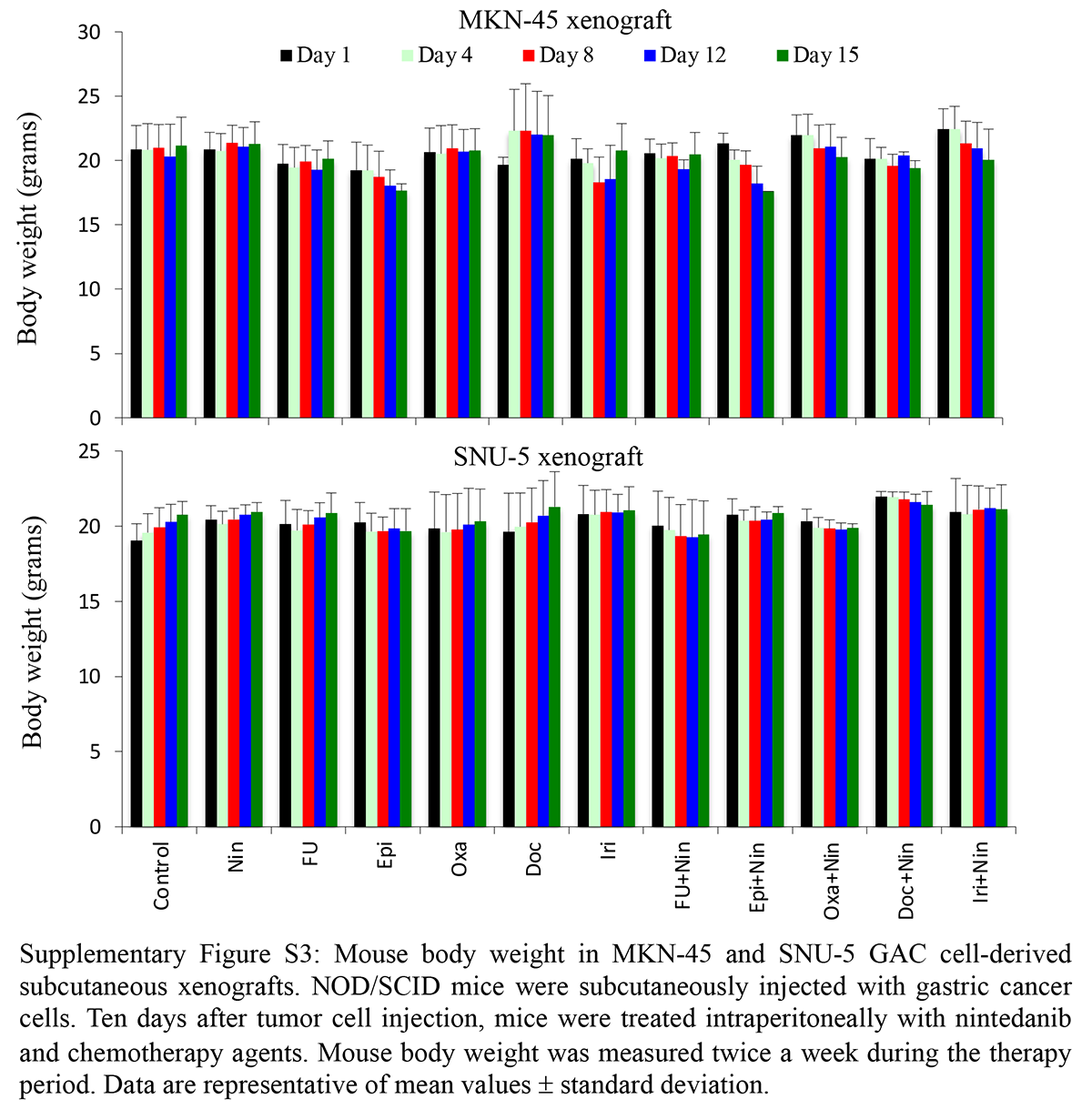

Supplement: Supplementary file 3 [file Image_3.tif]

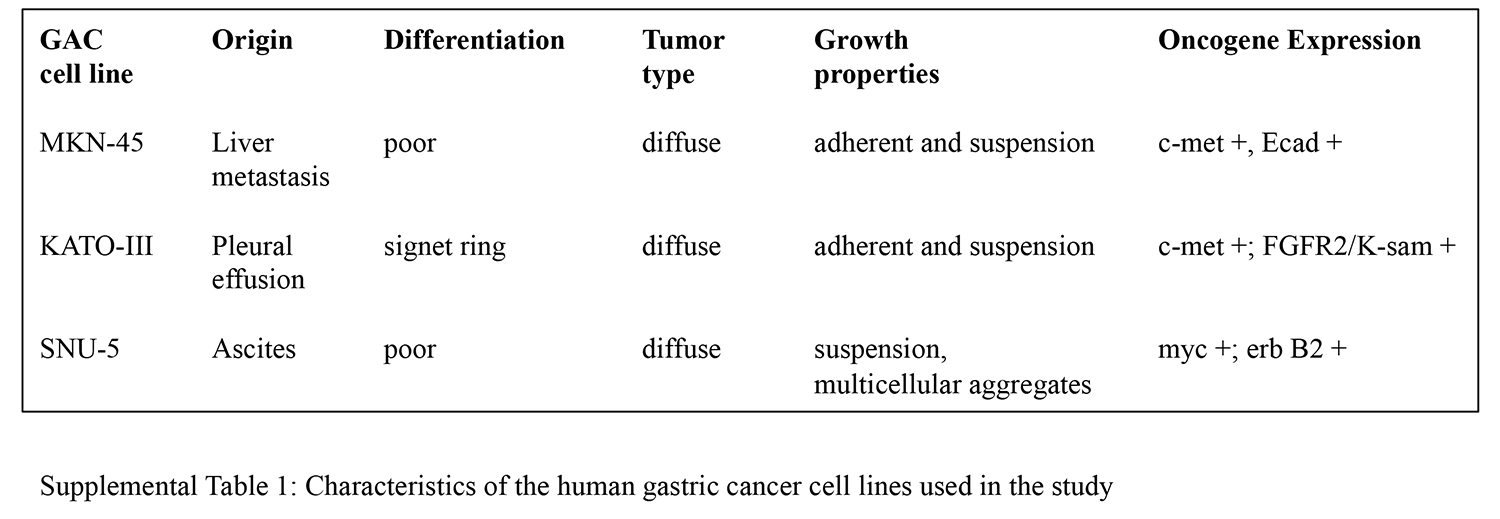

Supplement: Supplementary file 4 [file Image_4.tif]
